# Perfluorocarbon nanodrug induced oxygen self-enriching sonodynamic therapy improves cancer immunotherapy after insufficient radiofrequency ablation

**DOI:** 10.3389/fimmu.2023.1124152

**Published:** 2023-03-27

**Authors:** Tongyi Huang, Wenxin Wu, Jiancong Wu, Yang Tan, Minru Zhang, Haiyi Long, Huanling Guo, Xiaoer Zhang, Wenwen Zhou, Qi Zhang, Xiaoyan Xie, Ming Xu, Chunyang Zhang

**Affiliations:** ^1^ Department of Medical Ultrasonics, Institute of Diagnostic and Interventional Ultrasound, The First Affiliated Hospital of Sun Yat-sen University, Guangzhou, China; ^2^ School of Biomedical Engineering, Sun Yat-sen University, Guangzhou, China

**Keywords:** insufficient radiofrequency ablation, PD-1 blockade, oxygen self-enriching nanodrug, sonodynamic therapy, immunogenic cell death, antitumor immunity

## Abstract

Residual lesions and undetectable metastasis after insufficient radiofrequency ablation (iRFA) are associated with earlier new metastases and poor survival in cancer patients, for induced aggressive tumor phenotype and immunosuppression. Programmed cell death protein 1(PD-1) blockade has been reported to enhance the radiofrequency ablation-elicited antitumor immunity, but its ability to eliminate incompletely ablated residual lesions has been questioned. Here, we report a combined treatment modality post iRFA based on integrating an oxygen self-enriching nanodrug PFH-Ce6 liposome@O_2_ nanodroplets (PCL@O_2_)-augmented noninvasive sonodynamic therapy (SDT) with PD-1 blockade. PCL@O_2_ containing Ce6 as the sonosensitizer and PFH as O_2_ reservoir, was synthesized as an augmented SDT nanoplatform and showed increased ROS generation to raise effective apoptosis of tumor cells, which also exposed more calreticulin to induce stronger immunogenic cell death (ICD). Combining with PD-1 blockade post iRFA, this optimized SDT induced a better anti-tumor response in MC38 tumor bearing mouse model, which not only arrested residual primary tumor progression, but also inhibited the growth of distant tumor, therefore prolonging the survival. Profiling of immune populations within the tumor draining lymph nodes and tumors further revealed that combination therapy effectively induced ICD, and promoted the maturation of dendritic cells, tumor infiltration of T cells, as well as activation of cytotoxic T lymphocytes. While iRFA alone could result in an increase of regulatory T cells (Tregs) in the residual tumors, SDT plus PD-1 blockade post iRFA reduced the number of Tregs in both primary and distant tumors. Moreover, the combined treatment could significantly initiate long-term immune memory, manifesting as elevated levels of CD8^+^ and CD4^+^ central memory cells. Therefore, this study establishes the preclinical proof of concept to apply oxygen self-enriching SDT to augment cancer immunotherapy after iRFA.

## 1 Introduction

Radiofrequency ablation (RFA) is an effective curative thermal ablation treatment for tumors such as hepatocellular carcinomas, liver metastases, or renal cancers (1–3). Patients benefit from RFA treatment for its safety, minimal invasiveness, and effectiveness. RFA can also induce localized coagulation necrosis and release large amounts of cellular debris *in situ* to source tumor antigens to eliminate residual tumor cells (4, 5). However, in clinical practice, the clearance of the primary tumor by RFA alone sometimes can be difficult because of poorly defined tumor margin or undetected micrometastases, due to the limitation of tumor detection, or when tumors close to high-risk locations such as large blood vessels or bile ducts, lacking accessibility for complete RFA. Unfortunately, the RFA-induced immune responses are insufficient to eradicate residual tumors or prevent tumor recurrence. Similar to surgical resection of hepatocellular carcinomas, the 5-year recurrence rate of RFA treatment could be as high as 70%. It was reported that multiple mechanisms are involved and synergistically contribute to tumor recurrence after RFA. Several studies have shown that tumor tissue undergoes insufficient radiofrequency ablation (iRFA) at temperatures too low (42-60 °C) to kill residual tumor cells, leading to rapid and aggressive recurrence after iRFA (6, 7). iRFA is mainly due to poorly defined tumor margins or undetected micro-metastases, and can result in a post-RFA immunosuppressive microenvironment, which is crucial for further intervention. On the one hand, the iRFA can mediate local recruitment of tumor-associated macrophages and myeloid-derived suppressor cells to suppress immune surveillance (8). On the other hand, the presence of residual liver tumors was reported to associate with diminished response to immunotherapy, but not to targeted therapy or chemotherapy (9, 10). Because residual tumors can recruit immunosuppressive immune cells, including regulatory T cells (Tregs), to promote antigen-specific T cell apoptosis within the liver, thereby siphoning activated CD8+ T cells from systemic circulation. Thus, new adjuvant strategies are urgently needed to restrain iRFA-induced tumor cell progression.

Checkpoint inhibitor immunotherapy (CPI), especially programmed cell death protein 1/programmed cell death ligand 1 (PD-1/PD-L1) blockade, can reinvigorate the preexisting anti-tumor immunity to achieve a durable response rate, making it an important combination therapy in a post-RFA tumor recurrence. Wang et al. (11) reported that combination therapy with anti-PD-1 (aPD-1) plus RFA was superior to RFA alone in improving survival in patients with recurrent HCC, with 1-year recurrence-free survival rates of 32.5% and 10.0%. However, the response rate is still not high enough to prevent tumor recurrence, which may be compromised by the absence or exhaustion of CD8^+^ T cells, and an increase of immune-suppressive cells, such as Tregs. For instance, the iRFA-mediated local recruitment of Tregs suppressed immune surveillance and hindered PD-1 immunotherapy in colorectal cancer liver metastasis (10).

Recently, accumulating studies have shown that the immunosuppressive tumor microenvironment (TME) can be significantly modulated in ultrasound-triggered sonodynamic therapy (SDT), a noninvasive therapeutic strategy that can generate lethal reactive oxygen species (ROS) (12, 13). SDT applies sonosensitizers that are activated by low-intensity ultrasound (US) radiation to transfer energy to the surrounding oxygen (O_2_), generating ROS to directly kill or induce apoptosis of tumor cells, meanwhile exposing calreticulin (CRT) to induce immunogenic cell death (ICD), with minimal damage to healthy tissues. SDT induced ICD allows for maximum preservation of intact tumor antigens, which may trigger effective systemic immune responses to remove residual tumors and prevent tumor recurrence. Especially, most RFAs are performed under the guide of ultrasound in the clinical practice, which can be perfectly coordinated with SDT treatment to kill residual tumor cells.

**Figure 1:**
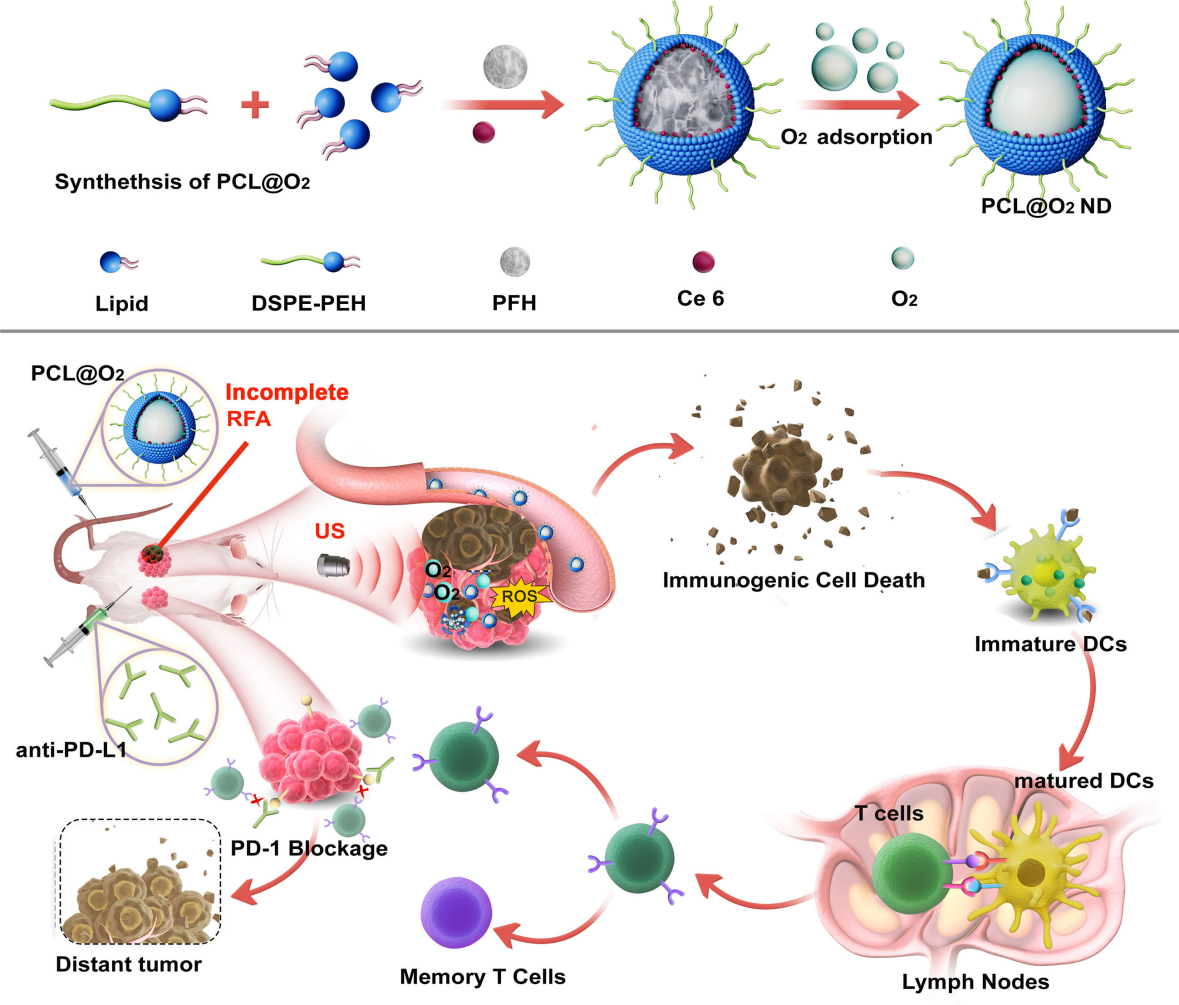
Schematic illustration of the synthesis of O_2_ loading PFH-Ce6 liposome@O_2_ nanodroplets abbreviated as PCL@O_2_ and *in vivo* antitumor performance of PCL@O_2_ in enhanced sonodynamic therapy (SDT)-immune combination therapy.

Herein, aiming to improve the immunotherapeutic efficacy and to reduce the tumor recurrence after RFA therapy, we designed a type of multifunctional nanodrug (PFH-Ce6 liposome@O_2_ nanodroplets, abbreviated as PCL@O_2_) to enhance the thermal ablation effect of RFA and improve the anti-PD-1 therapy after iRFA by encapsulating chlorine 6 (Ce6) as the sonosensitizers and phase-shifted perfluorohexane (PFH) liquid droplets as O_2_ reservoir, into liposomes (**Figure 1**). Under ultrasound irradiation, Ce6 can generate ROS for SDT and consume oxygen to aggravate the hypoxic microenvironment of the tumor. However, the hypoxic environment within the tumor is unfavorable for sonosensitizers that require O_2_ to exert their photocytotoxicity. Various strategies have been developed to overcome hypoxia, including the delivery of oxygen through artificial blood substitutes, such as perfluorocarbon, which has high affinity to O_2_ and good biocompatibility (14). The perfluorohexane (PFH) encapsuled in the nanodrug is a kind of perfluorocarbon with higher boiling points (around 58 °C), therefore having high *in-vivo* stability. PFH can enhance SDT not only by boosting the O_2_ level but also prolonging the lifetime of ROS inside tumor with its ROS storage capability, behaving like oxygen self-enriching (15). The further thermoelastic expansion of PFH can also enhance local tumor thermal ablation effects without significantly increasing the temperature (16). Furthermore, the PCL@O_2_ augmented SDT may induce effective ICD to active the T cell immune response, which could be promoted by the additional aPD-1 therapy. Thus, the SDT nanodrug (PCL@O_2_) may synergize with the aPD-1 treatment to elicit robust anti-cancer immunity and relieve immunosuppression of TME, providing potential strategies to eliminate residual tumors, enhance anti-tumor immunity, and reduce tumor recurrence after iRFA treatment.

## 2 Materials and methods

### 2.1 Materials

Dipalmitoyl phosphatidylcholine (DPPC), cholesterol (CHO), and 1,2-dioleoyl-sn-glycero-3-phosphoethanolamine-N-[methoxy(polyethyleneglycol)-2000 (DSPE-PEG-2000) were obtained from A.V.T. Pharmaceutical Co., Ltd. (Shanghai, China). Chlorin e6 (Ce6) and Perfluorohexane (PFH) were purchased from J&K Science Ltd. (Beijing, China). 1,3-Diphenylisobenzofuran (DPBF) was obtained from PYTHONBIO (Kaifeng, China), 2′,7′-Dichlorofluorescin diacetate (DCFH-DA) was obtained from Sigma-Aldrich. Anti-Calreticulin antibody was supplied by Abcam (Cat. #ab92516), Calcein AM was obtained from Beyotime (Cat. #C2012), PI was obtained from Solarbio (Beijing, China), CCK-8 was purchased from Dojindo Molecular Technologies, Inc. (Japan). The aPD-1 antibody was purchased from BioXcell (Cat. #BE0146). Antibodies for flow cytometry, including anti-CD45-PerCP (Cat. #103130), anti-CD3-FITC (Cat. #100204), anti-CD8-APC (Cat. #100711), anti-CD4-APC (Cat. #100412), anti-FoxP3-PE (Cat. #320008), anti-CD11c-FITC (Cat. #117306), anti-CD80-PE (Cat. #104708), anti-CD86-APC (Cat. #105012), anti-CD8-PE/Cy7 (Cat.# 100721), anti-CD4-BV650 (Cat. #100469), anti-CD44-PE (Cat. #103008), and anti-CD62L-APC (Cat. #104412) were obtained from Biolegend. Anti-CD8 antibody was supplied by Cell Signaling Technology (Cat. #98941S). Ki67 antibody was obtained from Servicebio Technology Co. Ltd., (Wuhan, China). ELISA kits were purchased from Dakewe Biotech Co., Ltd (Shenzhen, China).

### 2.2 Synthesis of nanodrug

The PCL@O_2_ was synthesized by filming-rehydration method. Briefly, A 6:3:1 weight ratio of DPPC:CHO: DSPE-PEG (total of 10 mg) and 1.0 mg Ce6 were dissolved in 2 mL CH_2_Cl_2_ in a 25 mL flask. Subsequently, the prepared film was hydrated and 100 μL of PFH was added into above suspension under sonication *via* a sonicator. Finally, PCL@O_2_ could be harvested by bubbling O_2_ gas in above PCL suspension. The resulted nanodroplets was stored at 4 °C. Ultimately, the Ce6 liposome@O_2_ nanodroplets (CL@O_2_) was prepared using the similar process except the addition of PFH.

### 2.3 Characterization of nanodrug

The Hydrodynamic diameter and Zeta potential of synthesized PCL@O_2_ were evaluated by *via* Nano ZS90 (Nano ZS, Malvern, USA). The morphology was obtained *via* transmission electron microscopy (TEM- JEM-2100F). These loading effect of Ce6 was evaluated by standard curve method *via* UV-vis spectrometer (Synergy HTX, BioTEK Co., USA).

### 2.4 *In vitro* ROS generation

Moreover, ROS generation of PCL@O_2_ triggered by ultrasonic irradiation was determined by DPBF, whose UV-via absorbance peak can be quenched by generated ROS. Briefly, DPBF (3 mM, 160 μL) and different formulation (PCL@O_2_, CL@O_2_ and H_2_O) were added into pre-deoxygenated water and the mixed solution (2 mL, Ce6 2.5 μg/mL, DPBF 240 μM) was subsequently irradiated by US (1.0 MHz, 1.6 W/cm^2^, 50% duty cycle) for 15 min. Then, the absorbance peak of DPBF was measured and recorded by UV–vis spectrophotometer.

### 2.5 *In vitro* O_2_ release

The O_2_ loading and ultrasound-responsive O_2_ release of oxygenated PCL@O_2_ were investigated by monitoring dissolved O_2_. Briefly, 1 mL different formulation (PCL@O_2_, CL@O_2_ and H_2_O) was added into 7 ml pre-deoxygenated water. After 2 min, the ultrasound irradiation (1.0 MHz, 1.6 W/cm^2^, 50% cycle) was carried out for 4 min. The concentration of dissolved O_2_ was detected in a sealed chamber equipped with portable dissolved oxygen meter (JPB-607A, INESA & Scientific Instrument Co., Ltd, Shanghai, China) in real time.

### 2.6 Cell lines

The murine colon adenocarcinoma cancer cell line MC38 was obtained from the Cell Bank of Shanghai Institute of Biochemistry and Cell Biology (China). The MC38 cell line was cultured in dulbecco’s modified eagle medium (DMEM, Gibco, USA) containing 10% fetal bovine serum (FBS, Gibco, USA) and 1% penicillin/streptomycin under 5% CO_2_ at 37 °C.

### 2.7 *In vitro* ROS detection

The intracellular ROS generation was evaluated by DCFH-DA. Briefly, after adhering in 24 wells plates, the MC38 cells were incubated with PCL@O_2_ or CL@O_2_ nanoparticles at Ce6 concentration of 5 μg/mL for 4 h. Subsequently, the cells were exposed to ultrasound irradiation (1.0 MHz, 1.6 W/cm^2^, 50% duty cycle) for 1 min, incubated with DCFH-DA (10 μM) for 30 min and then observed by fluorescence microscope (ECLIPSE TS2R FL, Nikon, Japan). Quantitative analysis of the fluorescence intensity was performed using Image J software.

### 2.8 *In vitro* cytotoxicity assessment

The cytotoxicity of SDT was evaluated by CCK-8 assay. After the completion of incubation and irradiation described previously, the cells were further incubated for another 2 h. The cell viability was assessed using CCK-8, and the OD 450 nm value was measured by a multimode reader (Synergy HTX, BioTEK Co., USA). Additionally, the cell viability was evaluated by live/dead cell staining. Similarly, the treated MC38 cells were stained with Calcein-AM and PI for 30 min, and observed by fluorescence microscope (ECLIPSE TS2R FL, Nikon, Japan). CRT expression on the treated cell surface was also observed to assess immunogenic cell death caused by SDT. Briefly, after fixation with 4% paraformaldehyde, the treated cells were blocked with 5% BSA, incubated with the anti-calreticulin antibody overnight and then with secondary antibody for 1 h. After further counterstaining with DAPI, the MC38 cells were visualized by fluorescence microscope. Semi-quantitative analysis of the fluorescence intensity was performed using Image J software.

### 2.9 *In vitro* dendritic cells maturation

Bone marrow cells were isolated from 6-week-old C57BL/6 mice and cultured according to the established protocol to induce immature bone-marrow-derived dendritic cells (BMDCs). The induced immature BMDCs were plated in the lower compartment of a transwell system (1×10^6^ cells per well). The MC38 cells treated with various treatments including incubation with nanodroplets for 4 h, irradiation to ultrasound (1.0 MHz, 1.6 W/cm^2^, 50% duty cycle, 1min) were put into the upper compartment of the transwell system. After co-incubation for 24 h, the treated BMDCs were stained with anti-CD11c-FITC, anti-CD80-PE and anti-CD86-APC and then collected for flow cytometric assessment.

### 2.10 Animal models and treatments

Female C57BL/6 mice (6-8 weeks old, 18-20 g) were provided by Guangdong Medical Laboratory Animal Centre. All animal experiments and operations were approved by Institutional Animal Care and Use Committee of the Sun Yat-sen University (approval number: SYSU-IACUC-2022-001655).

To develop bilateral subcutaneous MC38 tumor bearing mice, 40 μL and 120 μL of MC38 cell suspension (1×10^7^/mL PBS) were subcutaneously injected into the left and right flank, respectively. Treatments were initiated after 2 weeks, when the long diameter of the right tumor reached about 0.6 cm. The C57BL/6 mice bearing bilateral subcutaneous MC38 tumors were randomly divided into five groups receiving various treatments: (1) Control, (2) RFA, (3) RFA + aPD-1, (4) RFA + SDT, (5) RFA + SDT + aPD-1. The radiofrequency ablation treatments were performed using a radiofrequency electrode with an active tip of 1 cm. The tip was inserted percutaneously into the center of the right tumor for about 1 min depending on the actual tumor size, until the temperature reached 60°C within the tumor. For the SDT therapy, the PCL@O_2_ ([Ce6] = 667 μg/mL, 100 μg/injection) was intravenously injected three times on day 2, 5 and 8 after RFA administration. Subsequently, the primary tumor (the ablated one) of treated mice were irradiated with ultrasound (1.0 MHz, 1.6W/cm^2^, 30%, 8 min) at 12 and 24 h after PCL@O_2_ injection. For PD-1 blockade, 200 μg anti-PD-1 was administered through intraperitoneal injection every three days for a total of three times.

### 2.11 *In vivo* biodistribution of nanodrug

*In vivo* fluorescence imaging was performed to evaluate the accumulation of nanodroplets at tumor site and major organs. When the MC38 tumor volume grew up to 100 mm^3^, the *in vivo* fluorescence image of tumor-bearing mouse was acquired as control (0 h). After that, PIL@O_2_ (1 mg/kg IR-780) was intravenously injected *via* tail vein to reveal the biodistribution of nanodroplets. The fluorescence images were acquired at different time points (2, 4, 6, 9, 12, and 24 h) by Lumina XR Series III system (Perkin Elmer, USA, IR780: Ex, 720 nm; Em, 790 nm). 24 hours after the administration of PIL@O_2_, the major organs (heart, liver, spleen, lung and kidney) and tumor nodule were collected for *ex vivo* fluorescence imaging. Subsequently, the fluorescence signal intensities were quantified and calculated with living imaging system (IVIS lumina).

### 2.12 Tumor processing

Tumor diameters were measured using calipers every 2 days till 30 days after RFA treatment. The tumor volume was calculated using the formula, V = length × width^2^/2. For immune responses investigation, primary and distant tumors were harvested on the 10^th^ day and divided into three parts. A portion of the tumor tissues were minced and digested with 125 μg/mL collagenase and 12.5 μg/mL DNAse at 37 °C for 15 min, then ground and filtered with 1% FBS PBS buffer to prepare tumor cell suspension for flow cytometry analysis. A portion of the tumors were fixed in 4% paraformaldehyde for histology and the remaining tumors were prepared for tissue homogenates in PBS to analyze cytokines by Enzyme-linked immunosorbent assay (ELISA).

### 2.13 Flow cytometry analysis

The above-processed cell suspension was stained with fluorochrome-conjugated antibodies for the evaluation of CD8^+^ T cells (CD45^+^CD3^+^CD8^+^), CD4^+^ T cells (CD45^+^CD3^+^CD4^+^) and Tregs (CD45^+^CD3^+^CD4^+^FoxP3^+^) for 30min and then analyzed using flow cytometry. Tumor-draining lymph nodes were also collected and made into single-cell suspension to analyze dendritic cells (DCs, CD11c^+^CD80^+^CD86^+^) and central memory T cells (Tcm, CD44^+^CD62L^+^) in CD8^+^ T cells or CD4^+^ T cells.

### 2.14 Histology and immunofluorescence staining

Fixed tumor tissues in 4% paraformaldehyde were then embedded in paraffin, sectioned, dewaxed and rehydrated, and retrieved the antigen. Then, tumor tissue sections were conducted hematoxylin-eosin (H&E), and IHC staining by Ki67 and CD8. Eventually, staining images were obtained using microscope.

### 2.15 Measurements of cytokines

To further investigate the level of related cytokines (TNF-α and IFN-γ), the tumor tissue homogenates were assessed using the ELISA kits according to the instructions.

### 2.16 Biosafety evaluation

Major organs (heart, liver, spleen, lung, and kidney) from each group were obtained and fixed in 4% paraformaldehyde for H&E staining. Blood samples were collected, and the serological analyses containing hepatic and renal functional markers were carried out following the manufacturer’s protocol.

### 2.17 Statistical analysis

Prism 8.0 (GraphPad Software) was used to analyze data. Statistical analyses were conducted using two-tailed unpaired t test. Survival rates were analyzed by the Kaplan-Merier method and compared with log-rank test. Data were expressed as mean ± standard deviation (SD)/standard error of mean (SEM). *P* < 0.05 was considered to be statistically significant.

## 3 Results

### 3.1 Synthesis and characterization of nanodroplet

**Figure 2:**
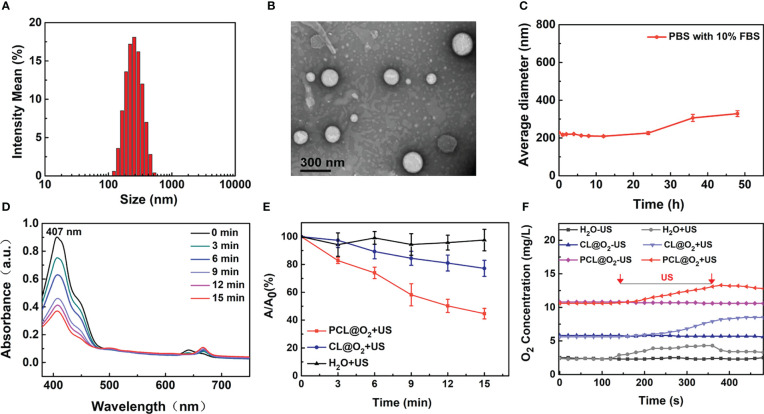
Characterization of the PCL@O_2_. **(A)** Hydrodynamic diameters of PCL@O_2_ measured by DLS. **(B)** Spherical morphology of PCL@O_2_ monitored by TEM. **(C)** Collodial stability of PCL@O_2_ dispersed in PBS containing 10% FBS. **(D)** UV-vis absorption spectra of DPBF in the presence of PCL@O_2_ under different ultrasound irradiation time (1.0 MHz, 1.6 W/cm^2^, 50% duty cycle). **(E)** Depletion effect of DPBF induced by differernt formulation under ultrasound irradiation (1.0 MHz, 1.6 W/cm^2^, 50% duty cycle, n = 3). **(F)** Dissolved oxygen concentrations of H_2_O, Ce6 liposome@O_2_ nanodroplets (CL@O_2_) and PCL@O_2_ nanodroplet solution under ultrasound irradiation (1.0 MHz, 1.6 W/cm^2^, 50% duty cycle). Data are presented as mean ± SD.

The SDT nanodroplet, PCL@O_2_, with a PFH core and a lipid membrane were synthesized by typical reverse evaporation method (**Figure 1**). Phospholipid as one of the most excellent biocompatibility materials was used as shell for loading hydrophobic agents Ce6 (sonosensitizer). Additionally, the O_2_-loaded nanodroplet, PCL@O_2_, was obtained by bubbling O_2_ gas. As light scattering (DLS) illustrated, PCL@O_2_ displayed an averaged hydrodynamic diameter of 230.17 ± 4.31 nm (PDI = 0.15 ± 0.02) (**Figure 2A**), and an averaged zeta potential of 0.47 ± 0.05 mV (**Supplementary Figure 1**). As monitored by the transmission electron microscopic (TEM) image (**Figure 2B**), the PCL@O_2_ showed a spherical morphology and homogeneous size. Additionally, the loading content of Ce6 was calculated to be 6.53 ± 0.05%.As shown in **Figure 2C**, the PCL@O_2_ nanodroplet was well dispersible in aqueous solution, and no aggregation in PBS containing 10% fetal calf serum (FBS) within 48 h.

To investigate the ROS production capacity of PCL@O_2_ under US irradiation, 1,3-diphenyliso- benzofuran (DPBF) were employed as molecular probe to monitor the ROS generation (13, 17). As shown in **Figures 2D, E**, the absorbance peak of DPBF in PCL@O_2_ solution decreased starkly under US irradiation, while the absorption peak of DPBF in CL@O_2_ decreased slowly. After US irradiation for 15 min, the decreased DPBF concentration of PCL@O_2_ group and CL@O_2_ nanodroplet was 55.3 ± 3.8% and 22.8 ± 5.8%, respectively. In comparison, the change of DPBF peak absorption in control group was negligible.

The O_2_-loaded PCL@O_2_ was synthesized as an augmented SDT nanoplatform. As reported, O_2_ can be loaded and stabilized by PFH for about two weeks, which significantly prolong the *in vivo* application time (18). As shown in **Figure 2F**, the dissolved oxygen concentration of PCL@O_2_ was obviously increased to 10.8 mg/L. In comparison, only a slight increase (up to 5.8 mg/L) of dissolved oxygen concentration was observed in CL@O_2_ group. After ultrasound irradiation, the PCL@O_2_-containing solution showed a dramatic increase of oxygen concentration to about 13.3 mg/L, which was higher than that of the CL@O_2_ group. Thus, results documented the good O_2_ loading ability and US-induced O_2_ release of PCL@O_2_, suggesting that PCL was potentially for delivering O_2_ to tumor *in vivo* and releasing it site-specifically under focal US irradiation.

### 3.2 *In vitro* ROS generation and SDT assessments

**Figure 3:**
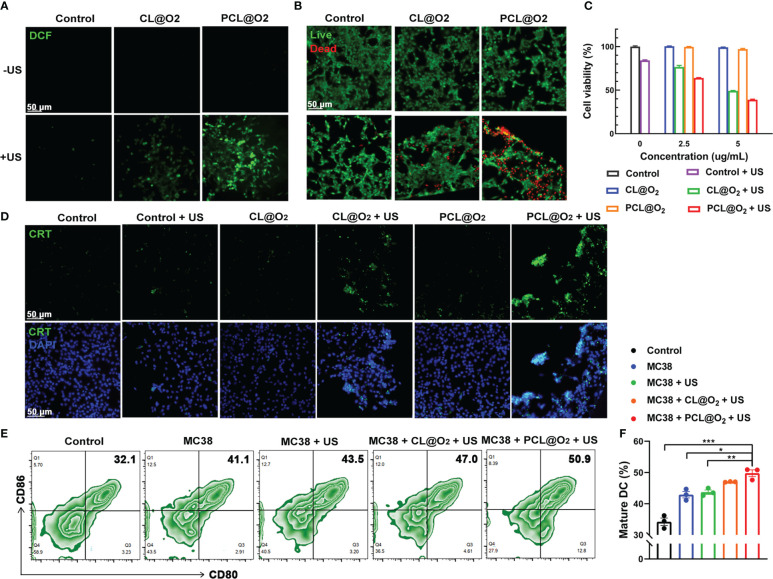
*In vitro* SDT assessments against MC38 cancer cells and maturation of DCs. **(A)** Intracellular ROS generation detection using DCFH-DA as a ROS probe after treatments with CL@O_2_ and PCL@O_2_ nanodroplets with or without ultrasound irradiation (1.0 MHz, 1.6 W/cm^2^, 50% duty cycle, 1 min). **(B)** Fluorescence images of MC38 cells stained with Calcein-AM (live cells, green fluorescence) and PI (dead cells, red fluorescence) after different treatments. **(C)** Viability of MC38 cells *via* CCK8 analysis. **(D)** Immunofluorescence staining of surface exposure of calreticulin (CRT) after various treatments. Scale bar: 50 μm. **(E)** Representative flow cytometric plots of mature dendritic cells (DCs, CD80^+^ CD86^+^ cells gating on CD11c^+^ cells) after 24 h co-incubation with treated MC38 cancer cells on the transwell system. **(F)** Proportion of mature DCs on the transwell system. Data are presented as mean ± SEM (n = 3). *P < 0.05, **P < 0.01 and ***P < 0.001.

The ROS generation inside MC38 cancer cells of Ce6-loaded nanodrug with US irradiation was measured with DCFH-DA, a ROS indicator. The Ce6 induced ROS with US irradiation can oxidate DCFH to strong fluorescent product DCF (oxidation product of DCFH), therefore showing the ROS level. As observed under confocal laser scanning microscopy, MC38 tumor cells incubated with PBS, CL@O_2_ and PCL@O_2_ without US irradiation or PBS with US irradiation showed negligible DCF fluorescence (**Figure 3A**, **Supplementary Figure 2A**), indicating US irradiation and sonosensitizer are both necessary for ROS generation. Compared with CL@O_2_, US irradiation induced higher green fluorescence intensity in cells incubated with PCL@O_2_, showing the robust synergy of O_2_-filled PFH in ROS generation. The live and dead cell viability assays were then carried out to evaluate the cytotoxicity effect of the generated ROS. Cells incubated with different nanodrugs without US irradiation or PBS with US irradiation showed strong green fluorescence, exhibiting good cell activity. In comparison with CL@O_2_ + US treatment, cells incubated with PCL@O_2_ + US showed much stronger red fluorescence and weaker green fluorescence, stating that PCL@O_2_ + US could result in more effective cytotoxicity (**Figure 3B**, **Supplementary Figure 2B**). ROS-induced cytotoxicity of nanodrug was further evaluated with CCK-8 analysis. Without US irradiation, cells incubated with PCL@O_2_(5 μg/mL) showed a good viability above 97%, indicating the nanodrug possessed a good biocompatibility. After irradiated with ultrasound, PCL@O_2_ + US at same concentration induced more apoptosis and a much lower viability (39%) than that of CL@O_2_ + US (49%) (**Figure 3C**).

Owing to SDT can generate intracellular ROS to induce ICD, immunofluorescence staining of CRT in MC38 cells, a common ICD marker that could stimulate antigen presentation and induce immune response (19, 20) was conducted. As shown in **Figure 3D** and **Supplementary Figure 2C**, cells treated with nanodrugs alone or US irradiation alone exhibited negligible green fluorescence. In comparison, the expression levels of CRT of PCL@O_2_ + US group was much stronger than that of CL@O_2_ + US. These results clearly proved that PCL@O_2_ induced enhanced SDT could significantly amplify ICD to potentially provoke an immune response.

### 3.3 Oxygen self-enriching SDT significantly improves maturation of DCs *in vitro*

To initiate tumor immunity, antigen presentation to cytotoxic T lymphocytes by antigen presenting cells (APCs) is indispensable, especially DCs. Activated DCs can capture tumor antigens and travel to lymphoid tissues where they activate tumor antigen-specific T lymphocytes. Maturation of DCs is a prerequisite for inducing effective anti-cancer immune responses. The ability of DCs to activate T cells correlates with their ability to mount anti-tumor immune responses (21, 22). Therefore, promoting the activation and maturation of DCs after RFA is quite critical.

In this study, transwell system was used to evaluate the maturation of DCs by culturing immature BMDCs with MC38 cells receiving various treatments. Because DCs start to mature upon antigen engagement, the differential effects on mature DCs may explain the effect of differential treatments on DCs *in vivo*. As shown in **Figure 3E**, oxygen self-enriching SDT by PCL@O_2_ + US clearly increased the number of CD11c^+^CD80^+^CD86^+^ mature BMDCs compared to other groups. The above results illustrated that the robust effect on DCs maturation may be due to that SDT promoted cancer cell death through apoptosis and necrosis, and DCs engulfed stressed and necrotic tumor cells.

### 3.4 Biodistribution of PCL@O_2_ nanodrug *in vivo*

**Figure 4:**
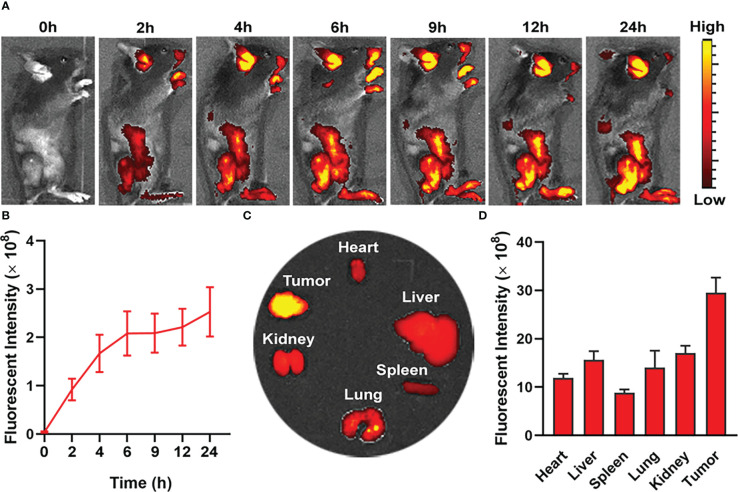
*In vivo* fluorescence imaging for biodistribution of nanodroplet in MC38 tumor-bearing mice. **(A)** *In vivo* fluorescence images of tumor bearing mouse showing biodistribution of nanodroplet at different time points after i.v. injection. **(B)** Quantification and variation of fluorescent intensities in tumor at different time points. **(C)** Fluorescence images and **(D)** fluorescence intensities of tumors and major organs *ex vivo* at 24 h after i.v. injection. Data are presented as mean ± SD.

The pharmacokinetics of nanodrug in MC38 tumor-bearing mice was evaluated *in vivo* by intravenous injection of PIL@O_2_ (1 mg/kg IR780). IR780 absorbs in the NIR region at 720 nm and can be captured with Lumina XR Series III system. The fluorescent intensities increased gradually post injection of nanodrug (**Figures 4A, B**). At the time point of 24 h, the tumor and major organs were collected, and fluorescence signal from IR780 was collected for quantification analysis. As shown in **Figures 4C** and **D**, the accumulation of nanodrug was obviously higher in the tumor than the other organs. This increased tumor accumulation may be promoted by enhanced permeability and retention (EPR) effect.

### 3.5 Synergistic anti-tumor effect of oxygen self-enriching SDT and immunotherapy in iRFA models

**Figure 5:**
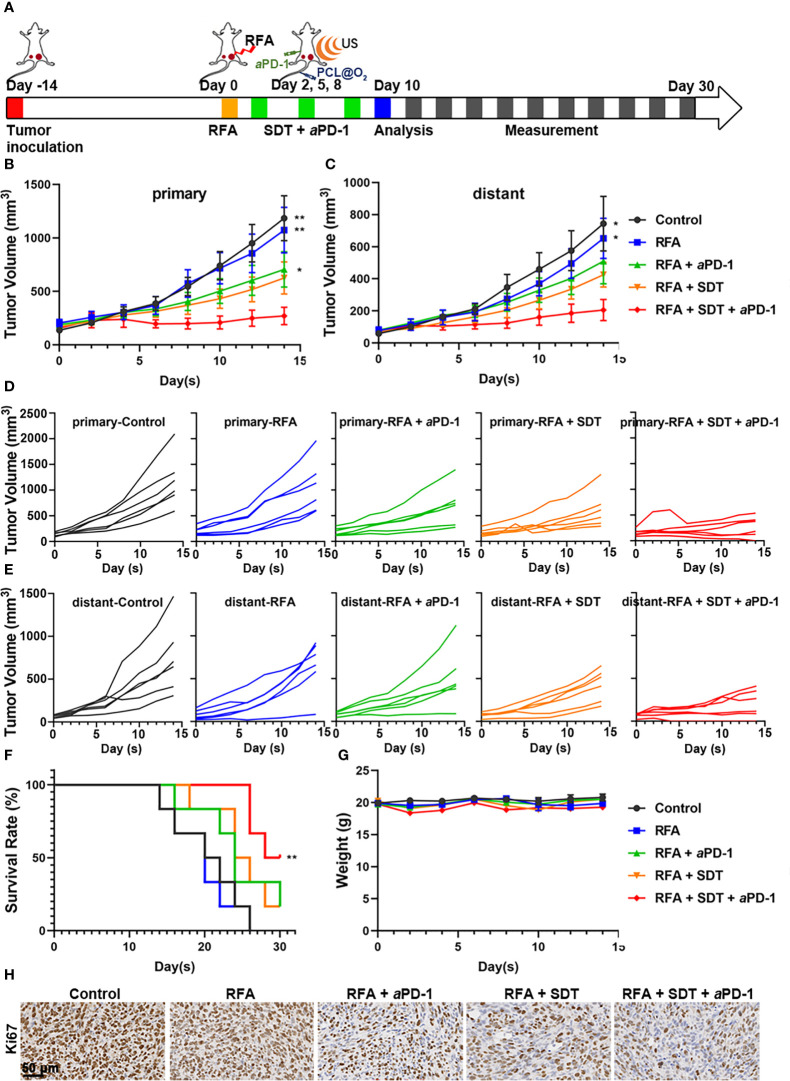
Synergistic anti-tumor effect of PCL@O_2_-enhanced SDT in combination with aPD-1 after incomplete radiofrequency ablation (RFA) in a bilateral subcutaneous tumor model. **(A)** Experimental design schematic for *in vivo* evaluation. After incomplete RFA of the right tumor (designed as the primary one), SDT (1.0 MHz, 1.6 W/cm^2^, 30% duty cycle, 8 min) was conducted three times after injecting nanodrug intravenously. The left tumor was set as the distant one without direct treatment. The aPD-1 was applied through intraperitoneal injection every three days for a total of three times. **(B)** Primary and **(C)** distant tumor volume curves of bilateral MC38 tumor-bearing mice receiving various treatments. **(D, E)** Individual tumor growth curves of **(D)** primary and **(E)** distant tumors. **(F)** The survival curves of MC38 tumor-bearing mice. **(G)** Body weight surveillance of mice receiving various treatments. **(H)** Representative immunohistochemical images of Ki67 in primary tumor sections at 2 d after various treatments. Scale bar: 50 μm. Data are presented as mean ± SEM (n = 6). *P < 0.05, **P < 0.01.

In further *in vivo* experiment, C57BL/6 mice were inoculated with MC38 on bilateral flanks, respectively, to evaluate the synergistic anticancer effect of SDT and anti-PD-1therapy *in vivo*. When the long diameter of the right tumor reached about 0.6 cm, the mice were randomly divided into the following five groups: control, RFA, RFA + aPD-1, RFA + SDT, and RFA + SDT + aPD-1. As shown in **Figure 5A**, insufficient RFA treatment was performed in the right tumor, which was defined as primary tumor, while the left tumor was defined as distant tumor. The volume of both primary and distant tumors in the RFA group of mice treated with iRFA was not significantly different from that of the control group, probably due to the smaller extent of ablation and the continued rapid growth of the residual tumors after ablation. All treatments with nanodrugs showed obvious therapeutic effects in both primary and distant tumors, and tumor growth was most effectively inhibited in mice treated with SDT and aPD-1 after iRFA. On day 10 after iRFA treatment, tumor volume of primary tumors in the RFA+ SDT+ aPD-1 group was 270.88 ± 82.06 mm^3^, and distant tumor reached 205.32 ± 66.13 mm^3^. In comparison, primary tumors grew to 1183.97 ± 209.82 mm^3^, 1073.08 ± 212.74 mm^3^, 706.02 ± 165.61 mm^3^ and 625.05 ± 150.44 mm^3^ in the control group, RFA group, RFA + aPD-1 group and RFA+SDT group, respectively (**Figures 5B, D**). A similar trend was observed for distant tumors, with tumor volume of 744.07 ± 170.24 mm^3^, 652.48 ± 125.56 mm^3^, 509.59 ± 140.65 mm^3^ and 425.53 ± 76.78 mm^3^ in the control group, RFA group, RFA + aPD-1 group and RFA + SDT group, respectively (**Figures 5C, E**). Consistent with the results of tumor growth inhibition, the RFA + SDT + aPD-1 treatment most effectively prolonged the survival rate of mice (**Figure 5F**). These results indicated that the combination of augmented SDT and aPD-1 raise a highly effective systemic anti-tumor immune response against both primary and distant tumor even with iRFA burdens, compared with the slightly inhibition of aPD-1 therapy alone.

To further validate the synergistic therapeutic efficacy, H&E, Ki-67 and TUNEL staining assays were performed on day 10 after iRFA in the five groups. As shown in **Supplementary Figure 3**, compact tumor cells with an intact structure were showed in the control group, and necrosis with destructive cells was observed in the groups treated with iRFA, while there were still surviving tumor cells around the ablation zone in RFA group. The results of TUNEL staining were consistent with the results of H&E staining. These results were further confirmed by the proliferation biomarker Ki-67 in iRFA zone of tumors (**Figure 5H**). Ki-67 was considerably reduced in each treatment group compared with control group, and significantly abridged by combined therapy of SDT and aPD-1. Therefore, combination therapy is superior to treatment alone in limiting cell proliferation in MC38 tumors after iRFA.

Additionally, the biosafety of PCL@O_2_ nanodrug was evaluated through body weight changes, H&E staining of the major organs, and blood biochemical markers. No significant changes in the body weights of the mice in different groups were observed after treatments (**Figure 5G**), and no obvious tissue damage or inflammation in heart, liver, spleen, lung, and kidney appeared in mice receiving different treatments (**Supplementary Figure 4**). In addition, the levels of alanine aminotransferase (ALT), alkaline phosphatase (ALP) and total bilirubin (TBIL) indicating the liver functions, as well as creatinine (CREA) and UREA indicating the kidney functions, were not elevated and within the normal range in mice receiving different treatments (**Supplementary Figure 5**), indicating that combination therapy did not injure the liver and kidney.

### 3.6 Optimized SDT, in combination with aPD-1 attracts and activates T cells to enhance anti-tumor immunity after iRFA

To further investigate the influence on immune microenvironment after different treatments from different angles, we collected tumor-draining lymph nodes, primary and distant tumors on the 10th day after first treatment for immunofluorescence staining, flow cytometry and cytokine analyses. Immunofluorescence staining of CRT in primary tumors was firstly conducted. As shown in **Supplementary Figure 6**, CRT staining on the cell membrane was clearly observed in combination treatment group, indicating PCL@O_2_ with enhanced SDT plus aPD-1 therapy could amplify ICD by CRT expression to potentially provoke an immune response after iRFA.

**Figure 6:**
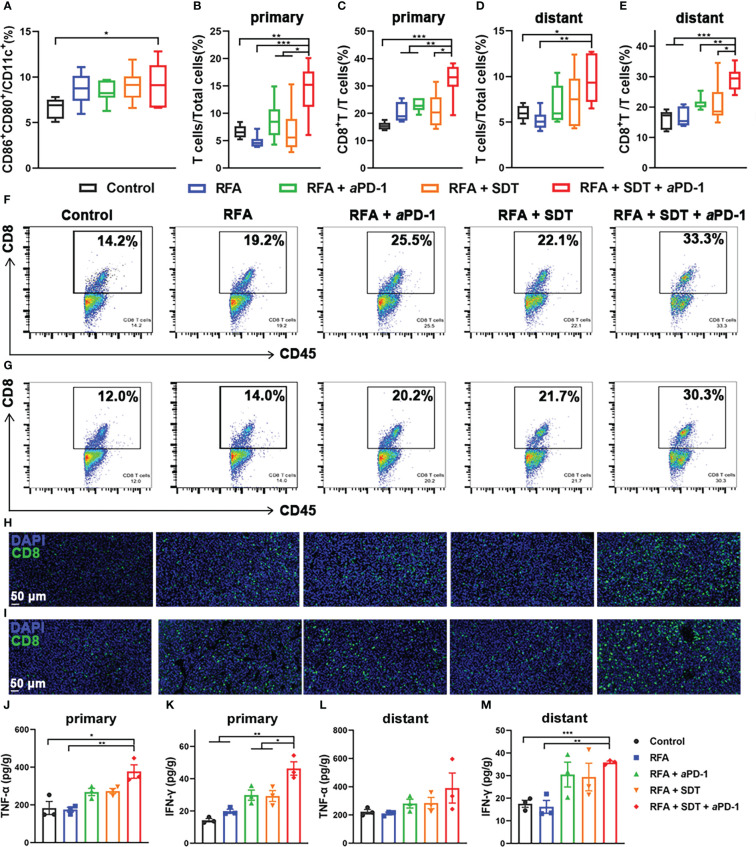
Systematic antitumor immunity caused by PCL@O_2_-augmented SDT plus aPD-1 therapy after incomplete RFA in MC38 tumor-bearing mice. **(A)** Proportion of mature DCs (CD80^+^CD86^+^) over CD11c^+^ DCs in tumor-draining lymph nodes by flow cytometry (n = 6). **(B–E)** T cells (CD45^+^CD3^+^): total cells ratios and CD8^+^ T cells (CD45^+^CD3^+^CD8^+^): T cells ratios in **(B, C)** primary tumors and **(D, E)** distant tumors after different treatments (n = 6). **(F, G)** The representative flow cytometric plots of CD8^+^ T cells in **(F)** primary and **(G)** distant tumors. **(H, I)** Immunofluorescence images of CD8+ T cells in **(H)** primary and **(I)** distant tumors (scale bar: 50 μm). **(J–M)** Cytokine levels of TNF-α and IFN-γ in **(J, K)** primary and **(L, M)** distant tumors receiving various treatments (n = 3). Statistical analyses were conducted using two-tailed unpaired t test. Data are presented as mean ± SEM. *P < 0.05, **P < 0.01 and ***P < 0.001.

Chronic exposure of damage-associated molecular patterns (DAMPs) by ICD could attract receptors and ligands on DCs and activate immature DCs to transition to a mature phenotype (19). Therefore, the maturation of DCs (CD11c^+^CD80^+^CD86^+^) in tumor draining lymph nodes was analyzed by flow cytometry after different treatment. As shown in **Figure 6A**, owing to the enhancement of SDT and aPD-1, the percentage of DCs maturation in RFA + SDT + aPD-1 group was 9.2%, which was significantly higher than that of control group (6.6%). Flow cytometry gating scheme for immune cells are described in **Supplementary Figure 7**.

Then, the percentage of tumor-infiltrating mature T cells (CD45^+^CD3^+^) in various treatment groups were measured, because tumor infiltration of T cells plays a key role in anti-tumor immunity. As shown in **Figures 6B** and **D**, the percentage of T cells in RFA + SDT + aPD-1 group obviously increased to 14.4 ± 1.9% and 9.6 ± 1.6% in primary and distant tumors respectively, which was nearly 2.2-fold and 1.6-fold higher than control group respectively. The percentage of T cells in combination therapy group was also significantly higher than RFA, RFA + aPD-1 and RFA + SDT group. A similar trend was seen in the percentage of CD8^+^ cytotoxic T lymphocytes (CTLs). As shown in **Figures 6C** and **F**, the SDT plus aPD-1 treatment after iRFA resulted in a much higher percentages of CTLs in T cells (32.3 ± 2.7%) than treatments using RFA alone (20.2 ± 1.4%), RFA + aPD-1 (22.9 ± 1.0%), RFA + SDT (21.1 ± 2.6%), and PBS (15.4 ± 0.6%) in primary tumors. Consistent results were obtained in distant tumor tissues. The percentage of CTLs in combination treatment group (29.2 ± 1.6%) was significantly higher than other groups (**Figures 6E, G**). Tumor infiltration of the CD8^+^ T cells was further confirmed by immunofluorescent staining of tumor tissue sections. Comparison with the rare tumor infiltration of CD8^+^ T cells in the control group, CD8^+^ T cells was obviously increased *via* combination therapy in both primary and distant tumors (**Figures 6H, I**). In particular, the RFA + SDT + aPD-1 treatment showed the best effect to promote the tumor infiltration of CD8^+^ T cells. This may be due to increased mature DCs facilitating antigen presentation to induce more cytotoxic T lymphocytes (CTLs). These results showed that the oxygen self-enriching nanodrug may mediate effective Ce6-based SDT synergistic with aPD-1 treatment to enhance the tumor infiltration of CD8^+^ T cells.

Considering that TNF-α and IFN-γ produced by immune cells can induce the secretion of pro-inflammatory cytokines for a strong anti-tumor immunity (23, 24), the levels of TNF-α and IFN-γ in primary and distant tumors were also detected on the 10th day after first treatment by ELISA to verify the activation of the tumor-cell killing of immune cells. As shown in **Figures 6J–M**, compared with the control treatment or RFA alone, combination therapy of SDT and aPD-1 after iRFA induced a significant increase of both TNF-α and IFN-γ levels in bilateral tumors. Moreover, the level of IFN-γ induced by SDT plus aPD-1 therapy after iRFA was even much higher than that of the RFA + aPD-1 or RFA + SDT, which implied a strong synergistic effect of SDT and aPD-1 to activate T cells and initiate effective anti-tumor immune response after iRFA.

### 3.7 Combination therapy reverses immunosuppression of TME by eliminating Tregs

**Figure 7:**
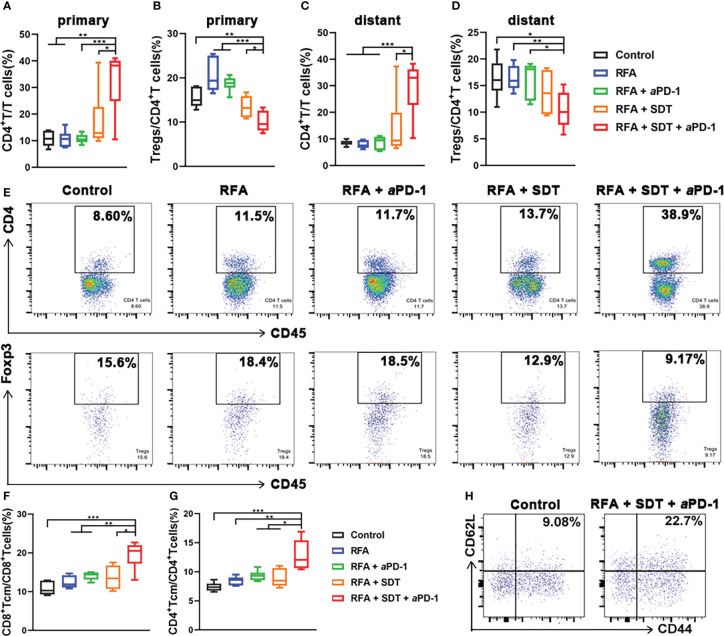
Systemic immune-modulating effects induced by PCL@O_2_-enhanced SDT and aPD-1 following incomplete RFA in MC38 tumor-bearing mice. **(A–D)** CD4^+^ T cells (CD45^+^CD3^+^CD4^+^): T cells (CD45^+^CD3^+^) ratios and regulatory T cells (Tregs, CD45^+^CD3^+^CD4^+^Foxp3^+^): CD4+ T cells ratios in **(A, B)** primary tumors and **(C, D)** distant tumors after different treatments by flow cytometry. **(E)** The representative flow cytometric plots of CD4^+^ T cells and Tregs in primary tumors. **(F, G)** Percentages of central memory T cells (Tcm, CD44^+^CD62L^+^) over **(F)** CD8^+^ T cells (CD3^+^CD8^+^) and **(G)** CD4^+^ T cells (CD3^+^CD4^+^) in tumor-draining lymph nodes measured by flow cytometry. **(H)** The representative flow cytometric plots of Tcm over CD8^+^ T cells in tumor-draining lymph nodes. Statistical analyses were conducted using two-tailed unpaired t test. Data are presented as mean ± SEM (n = 6). *P < 0.05, **P < 0.01 and ***P < 0.001.

The immune response to tumor antigen presence within the tumor, especially in the liver cancer, where the RFA therapy mostly applied, could lead to the systemic suppression of antitumor immunity (10). This immune suppression was associated with the coordinated activation of Tregs and modulation of intratumoral CD11b^+^ monocytes. Thus, the attraction of Tregs was characterized by the positive expression of both CD4 and Foxp3 using flow cytometry. As shown in **Figures 7A, B** and **E**, although there was an increase in total CD4 T cells in primary tumors of RFA + SDT + aPD-1 group, the proportion of Tregs in CD4 T cells significantly deceased in combination therapy group, compared to control, RFA alone, RFA + aPD-1 or RFA + SDT group. A similar trend also appeared in the distant tumor (**Figures 7C, D**, **Supplementary Figure 8**). It is worth noting that there was an increase of Tregs in primary tumor of RFA group, which indicating that insufficient RFA therapy could induce reduction in systemic antitumor immunity and restrain immunotherapy efficacy *via* Tregs-mediated immune suppression. Interestingly, this increase of Tregs after iRFA can be diminished by optimized SDT therapy and further eliminated by combination of aPD-1 therapy.

### 3.8 Combination therapy generates long-term memory to enhance systemic anti-tumor immunity

Exhaustion of effector T cells following tumor antigen clearance, which results in their short persistence, can limits the efficacy of immunotherapy after iRFA. Since the long-lived Tcm persist longer and produce stronger effector functions that have the potential to control tumor recurrence after RFA, their generation is an important goal for the combination treatment (25). Thus, the proportion of CD8^+^ Tcm (CD3^+^ CD8^+^ CD62L^+^ CD44^+^) and CD4^+^ Tcm (CD3^+^ CD4^+^ CD62L^+^ CD44^+^) in tumor-draining lymph nodes was measured to explore the long-term immune memory effect. As shown in **Figures 7F** and **H**, the RFA + SDT + aPD-1 combination therapy resulted in a significant increase in CD8^+^ Tcm, compared with the treatment of control, RFA alone, RFA + SDT or RFA + aPD-1. Interestingly, the proportion of CD4^+^ Tcm also obviously increased in the RFA + SDT + aPD-1 group (**Figure 7G**), which may also produce more antigen-specific CD4^+^ T cells. These antigen-specific CD4^+^ T cells are reported to enhance the efficacy of anti-tumor CD8^+^ T cell responses, and mediate long-lived protection against subsequent tumor recurrence (26). These results indicated that the synergistic SDT and aPD-1 therapy after iRFA can elicit effective and long lasting anti-tumor response.

## 4 Discussion

RFA, a common locoregional curative therapy, has been widely used in tumor therapy, especially in hepatocellular carcinomas and liver metastases, for its minimal invasiveness and efficient tumor-killing ability. In clinical practice, the clearance of the primary tumor by RFA alone sometimes can be difficult because of poorly defined tumor margin or undetected micrometastases, due to the limitation of tumor detection, or when tumors close to high-risk locations such as large blood vessels or bile ducts, lacking accessibility for complete RFA. Thus, residual lesions and undetectable metastasis after iRFA are major causes of tumor recurrence and treatment failure in cancer patients with RFA treatment (7). In theory, iRFA could also promote the release of tumor antigens by causing massive cell death to induce anti-tumor immunity for further tumor cell clearance. Unfortunately, this advantage is undermined by the immunosuppressive microenvironment following RFA, which is mainly due to the exhaustion of T cells and the accumulation of immune cells with immunosuppressive tendencies, such as increased Tregs (8). Further combined treatment modalities are urgently needed to solve this problem. The PD-L1/PD-1 blockade has become a central issue for cancer immunotherapy for its advantage in improving the survival rates of patients with various types of hematologic and solid malignancies. RFA combined with PD-1/PD-L1 blockade immunotherapy has been proven to be effective in preclinical and some clinical studies (8, 27). However, most patients do not benefit much from this treatment, including patients with incomplete RFA. In fact, the iRFA-induced local recruitment of Tregs, monocytes and tumor associated macrophages can inhibit T cell functionality, promote tumor progression, and hinder the efficacy of anti-PD-1 therapy in colorectal cancer liver metastases (8). In this study, the iRFA also induced an increase of Tregs in the primary tumors as shown in **Figure 7B**. Therefore, further synergistic therapy should be investigated to induce stronger anti-tumor immunity and enhance PD-L1/PD-1 blockade therapy post iRFA.

Unlike direct thermal ablation, SDT uses US activated sonosensitizers to transfer energy to the surrounding O_2_, generating ROS to directly kill or induce apoptosis of tumor cells, which can expose CRT to induce ICD. SDT induced ICD allows for maximum preservation of intact tumor antigens, that can trigger effective systemic immune responses to inhibit distant metastatic tumors and prevent tumor recurrence. Considering most RFAs are guided by ultrasound in the clinical practice, the SDT can just apply the minimally invasive deep tissue penetration of ultrasound and can avoid unnecessary thermal damage to healthy structures (for example, bile ducts or large vessels). Therefore, SDT is an ideal complementary treatment to RFA and can provide a salvage alternative for residual tumors after iRFA.

In this study, a nanodrug of PCL@O_2_ containing Ce6 as the sonosensitizer and PFH as O_2_ reservoir, was synthesized as an augmented SDT nanoplatform and showed excellent ROS production capacity. Due to the dependence of SDT on oxygen, its efficacy is often compromised by the pre-existing hypoxia of the tumor and the continuous oxygen consumption (28). In the PCL@O_2_ nanodrug mediated SDT platform, oxygen could be loaded and stabilized by PFH for increased ROS generation, behaving like oxygen self-enriching. As shown before, the dissolved oxygen concentration of PCL@O_2_ group was nearly 2-fold higher than CL@O_2_ group.

Our results showed that PCL@O_2_ induced sonodynamic therapy is significantly enhanced compared with traditional SDT in the tumor matrix *in vitro*. With US irradiation, the *in vivo* studies showed more ROS was generated to better damage and kill MC38 cancer cells by PCL@O_2_, compared to the PBS and traditional SDT agent CL@O_2_, suggesting PFH can help the sonosensitizer achieve improved effects. The increased ROS by PCL@O_2_ + US naturally induced stronger immunogenic cell death, which was proved by CRT staining.

Ablation of the tumor itself can induce ICD, thus providing a vaccine effect. The destruction of tumor cells during this process leads to the production and secretion of tumor-specific antigens. Antigens are taken up by dendritic cells in the presence of immunostimulatory cytokines, leading to the initiation of an adaptive immune response by T cells. The degree of tumor destruction, such as complete versus incomplete ablation, can also influence the antitumor immune response. In humans and preclinical models, incomplete RFA may elicit suppressive immune responses. Incomplete ablation may lead to an uneven degree of cell death between treatment groups with different levels of antigen and immune stimulation, thus given its potential to evoke different death pathways and different levels of immunogenicity and ICD. For example, Xiaoqiang Qi et al. reported that iRFA was able to directly damage tumors by inducing tumor necrosis and apoptosis that is considered more physiological, modifiable, and non-immune, and therefore has a lower immunogenicity (19, 29). The increase in immunosuppressive mechanisms, without a significant difference in the frequency of T cells, may indicate that incomplete ablation promotes only a slight degree of ICD. In contrast, in our study, PCL@O_2_-enhanced SDT induced homogeneous and potent ICD, just complementary to incomplete ablation in the absence of a sufficiently effective ICD.

At the same time, the activation and maturation of DCs typically starts when DCs identify ICD signals. However, DCs function could be suppressed by tumor-mediated microenvironment after iRFA. Our result showed that the oxygen self-enriching SDT significantly improved maturation of DCs *in vitro*. It is reasonable to speculate that the improved DCs maturation by oxygen self-enriching SDT would mask some of the *in vivo* immunosuppressive effects of iRFA. In summary, our studies suggested that synergistic SDT to improve DCs maturation is a highly effective strategy to enhance immune response of iRFA.

PD-1 blockade has been reported to enhance the RFA-elicited antitumor immunity, but its ability to clear residual incompletely ablated lesions has been questioned (8). In this study, treatment with PD-1 mAbs alone had little effect in combination with iRFA, and no significant differences were found in the growth curve or survival in both residual tumors and distant tumors, compared with the RFA group. These results indicated that both residual primary tumors and distant tumors after iRFA are resistant to anti-PD-1 therapy. In contrast, with intravenous injection into tumor-bearing mice, PCL@O_2_ induced SDT showed obvious synergistic effect with aPD-1 therapy post iRFA and exhibited significant tumor growth inhibition, as well as prolonged survival time, whereas SDT or aPD-1 alone showed negligible tumor inhibition and survival improvement. It should be noted that improved anti-tumor immunity was supposed to play an important role in these synergistic treatment effect. As with the primary tumors, in the secondary tumors without direct SDT treatment, RFA + SDT + aPD-1 also resulted in best tumor growth inhibition, compared to other treatments.

To understand how combination therapy remodeled the tumor microenvironment, profiling of immune populations within the tumor draining lymph nodes and tumors were performed and revealed that PCL@O_2_ nanodrug mediated SDT plus aPD-1 therapy effectively induced immunogenic cell death, promoted the maturation of DCs, tumor infiltration of T cells, and activation of CTL (**Figure 6**). Interestingly, we found iRFA alone could result in an increase of Tregs in the residual tumors. This result raised the possibility that residual tumor cells post iRFA might trigger Tregs-dependent tolerance mechanisms that reduce systemic antitumor immunity and cancer immunotherapy efficacy. James C et al. elucidated that anti-PD-1 treatment failure was associated with the coordinated activation of Tregs in liver metastasis, by down-regulating or striping CD80/86 from APCs, preventing CD28-mediated costimulation of antigen-specific effector T cells (10). In contrast, SDT + aPD-1 markedly reduced the number of Tregs in both primary and secondary tumors post iRFA. Studies has shown that the hypoxia inside the tumor microenvironment can promote FoxP3 expression or impact the cytokine profile inside to attract Tregs (30, 31). The oxygen self-enriching ability of our nanodrug may alleviate tumor hypoxia to reduce Tregs. Therefore, our results support the rationale that Tregs-targeted combination immunotherapy can be further deployed more precisely in cases where Tregs activity may be enhanced by incomplete ablation.

Analysis of cytokine expression in tumors also provides an opportunity to propose pharmacological interventions for optimizing antitumor immunity by mitigating or manipulating cytokine interference. Certain antitumor cytokines including TNF-α and IFN-γ are secreted for several hours after ablation and decrease rapidly (32). This suggests that the use of checkpoint inhibitors or other immunomodulators may be an effective tool in combination with ablation to maintain higher levels of antitumor cytokines for a longer period. In our study, compared with other group, combination therapy of SDT and aPD-1 after RFA induced a significant increase of both TNF-α and IFN-γ levels in bilateral tumors. PCL@O_2_ nanodrug-mediated SDT plus aPD-1 therapy promoted the secretion of antitumor cytokines after RFA, which further supports the strong synergistic effect of SDT and aPD-1 on activating T cells and initiating an effective antitumor immune response after iRFA.

The efficacy of combination therapy depends not only on its ability to effectively eradicate existing tumors, but also on preventing the recurrence of tumors. Our data suggested that SDT plus aPD-1 induced anti-tumor immunity post iRFA could significantly prolong survival and initiate long-term immune memory, effectively preventing tumor recurrence. This immunity manifested as elevated levels of memory T cells, including central memory cells of CD8^+^ and CD4^+^ lineages. Based on these results, we hypothesize that PCL@O_2_ enhanced SDT will be able to enhance immunotherapy after incomplete ablation in clinical applications, providing a salvage alternative for residual and recurrent tumors and associated immune tolerance after ablation.

## 5 Conclusion

In summary, residual tumors post iRFA might lead to rapid tumor progression and immunosuppression, thereby limiting the efficacy of PD-1 blockade therapy. It is necessary to investigate a further synergistic therapy to eliminate residual tumors and induce stronger anti-tumor immunity. O_2_ reservoir PFH-containing PCL@O_2_ nanodrug mediated SDT can use US activated sonosensitizers Ce6 to transfer energy to the surrounding O_2_, generating more ROS to induce apoptosis of tumor cells, for its oxygen self-enriching ability. This enhanced SDT induced stronger immunogenic cell death, which worked synergistically with PD-1 blockade to inhibit residual tumors and metastases after iRFA. This combination of augmented SDT and PD-1 blockade post RFA was able to promote the maturation of DCs, tumor infiltration of T cells, activation of CTLs, and inhibition of Tregs, for improving antitumor immune response. Furthermore, sustained antitumor memory response was built to prevent tumor recurrence. This study established the preclinical proof of concept to apply ultrasonography-guided oxygen self-enriching SDT to augment cancer immunotherapy after iRFA of solid tumors.

## Data availability statement

The raw data supporting the conclusions of this article will be made available by the authors, without undue reservation.

## Ethics statement

The animal study was reviewed and approved by Institutional Animal Care and Use Committee of the Sun Yat-sen University (approval number: SYSU-IACUC-2022-001655).

## Author contributions

Conception and design, CZ, MX, XX, TH, WW, YT, and JW. Performed the experiments, CZ, TH, WW, JW, MZ, WZ, HL, HG, and QZ. Data analysis and presentation, TH, CZ, MX, XZ, and WW. Manuscript writing, TH, CZ, and WW. Final approval of manuscript, all authors. All authors contributed to the article and approved the submitted version.

## Supplementary material

Supplementary Figure 1 Zeta potential of PFH-Ce6 liposome@O_2_ nanodroplets (PCL@O_2_) measured by DLS.

Supplementary Figure 2 Semi-quantification of the fluorescence intensity of **(A)** DCF, **(B)** live/dead cells and **(C)** calreticulin (CRT) against MC38 cancer cells after treatments with nanodroplets and ultrasound irradiation (1.0 MHz, 1.6 W/cm^2^, 50% duty cycle, 1 min), as shown in Figures 3A, B and D. Data are presented as mean ± SEM (n = 3).

Supplementary Figure 3 HE staining and TUNEL immunofluorescence staining of ablated tumor tissue. White line marked out the border between ablated and non-ablated tissue, scale bar: 200 μm.

Supplementary Figure 4
*Ex vivo* pathological H&E staining of the heart, liver, spleen, lung, and kidney of mice receiving different treatments. The major organs were collected and examined on day 10 after the first treatment. Nuclei were stained blue, while cytoplasm and extracellular matrix were stained red in H&E staining, scale bar = 200 μm.

Supplementary Figure 5 Analyses of serum function markers of the liver (ALT, ALP and TBIL) and kidney (CREA and UREA). Data are presented as mean ± SEM (n = 6).

Supplementary Figure 6 Immunofluorescence images of CRT in primary tumors of the control group and combination treatment group (scale bar: 50 μm).

Supplementary Figure 7 Gating strategies for flow cytometry analyses of immune cells. **(A)** Gating strategy to determine the percentage of mature DCs in CD11c^+^ DCs, as shown in Figure 5A. **(B)** Gating strategy to determine the percentage of T subsets in CD45^+^ immune cells, as displayed in Figures 5B-G and Figures 6A-E. **(C)** Gating strategy to determine the percentage of Tcm in CD8^+^ T cells or CD4^+^ T cells, as shown in Figures 6F-H.

Supplementary Figure 8 The representative flow cytometric plots of CD4^+^ T cells and Tregs in distant tumors receiving different treatments.
