# Supplementary figures and images for: Perfluorocarbon nanodrug induced oxygen self-enriching sonodynamic therapy improves cancer immunotherapy after insufficient radiofrequency ablation
Source: Front Immunol. 2023 Mar 27;14:1124152. doi: 10.3389/fimmu.2023.1124152 (PMC10083362; doi:10.3389/fimmu.2023.1124152)

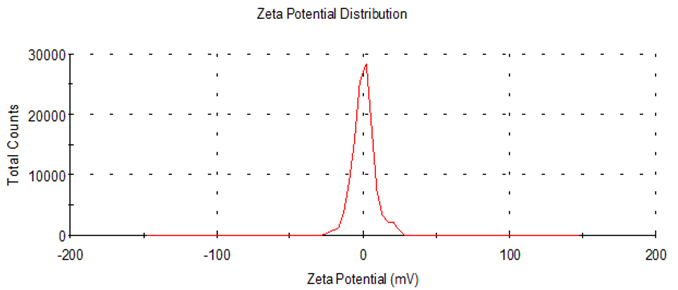

Supplement: Supplementary Figure 1 — Zeta potential of PFH-Ce6 liposome@O2 nanodroplets (PCL@O2) measured by DLS. [file Image_1.png]

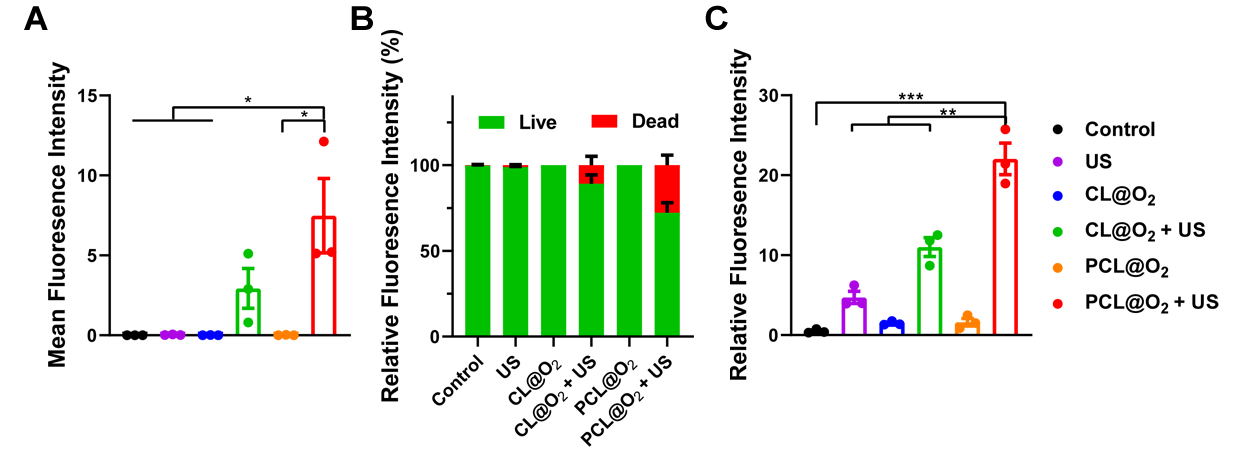

Supplement: Supplementary Figure 2 — Semi-quantification of the fluorescence intensity of (A) DCF, (B) live/dead cells and (C) calreticulin (CRT) against MC38 cancer cells after treatments with nanodroplets and ultrasound irradiation (1.0 MHz, 1.6 W/cm2, 50% duty cycle, 1 min), as shown in Figures 3A, B and D. Data are presented as mean ± SEM (n = 3). [file Image_2.tif]

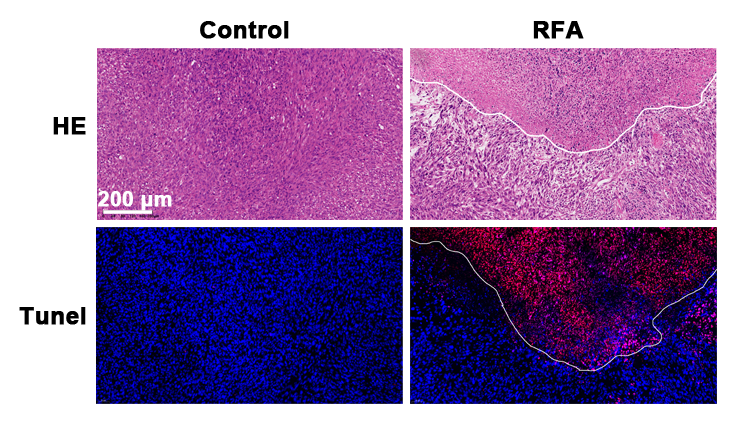

Supplement: Supplementary Figure 3 — HE staining and TUNEL immunofluorescence staining of ablated tumor tissue. White line marked out the border between ablated and non-ablated tissue, scale bar: 200 μm. [file Image_3.tif]

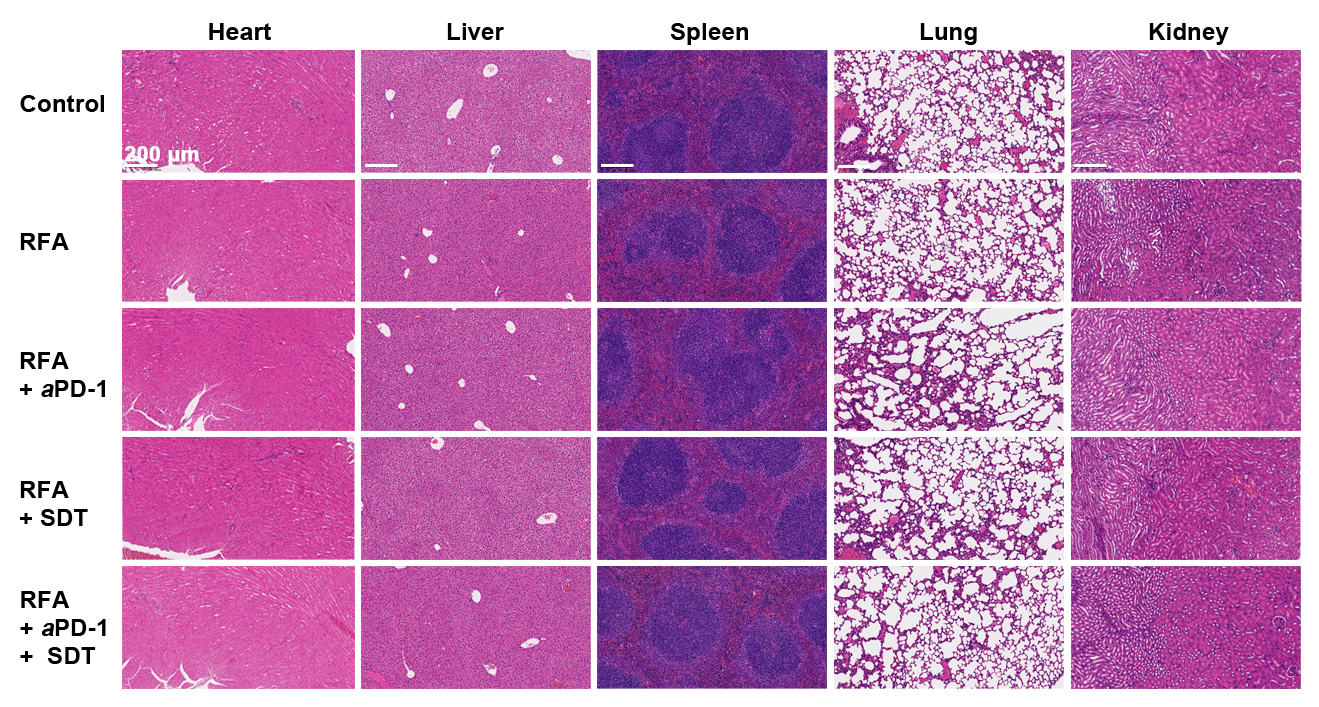

Supplement: Supplementary Figure 4 — Ex vivo pathological H&E staining of the heart, liver, spleen, lung, and kidney of mice receiving different treatments. The major organs were collected and examined on day 10 after the first treatment. Nuclei were stained blue, while cytoplasm and extracellular matrix were stained red in H&E staining, scale bar = 200 μm. [file Image_4.tif]

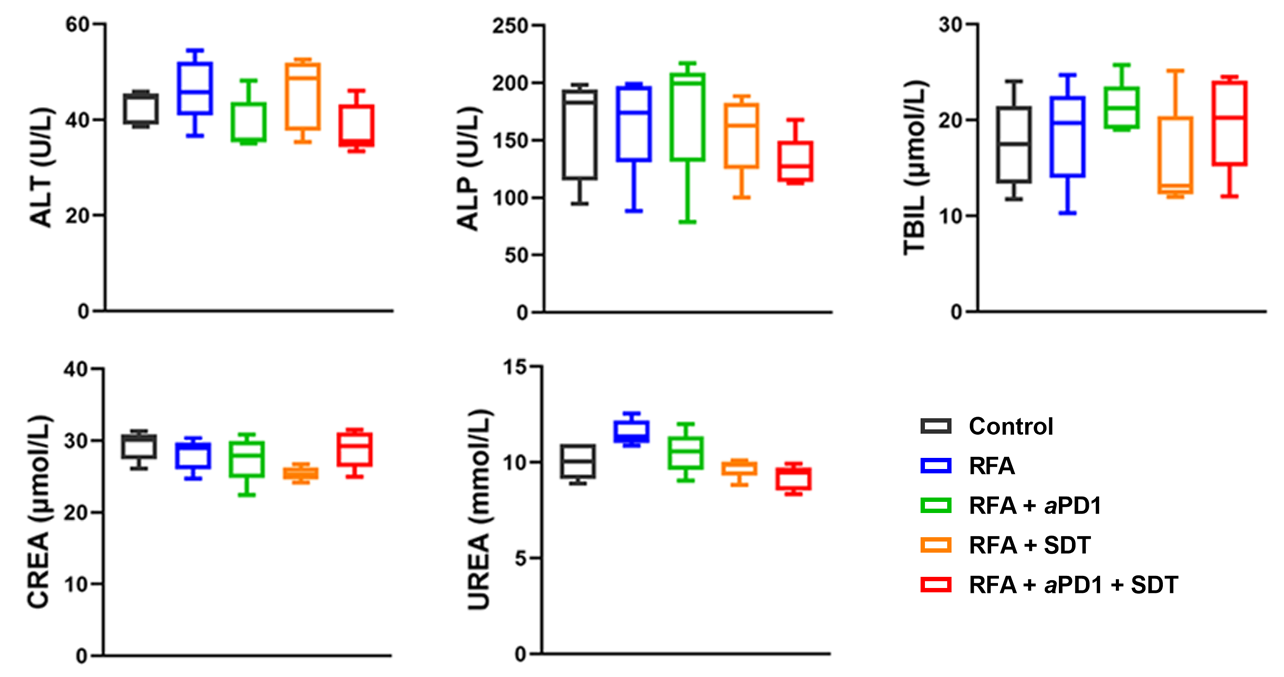

Supplement: Supplementary Figure 5 — Analyses of serum function markers of the liver (ALT, ALP and TBIL) and kidney (CREA and UREA). Data are presented as mean ± SEM (n = 6). [file Image_5.tif]

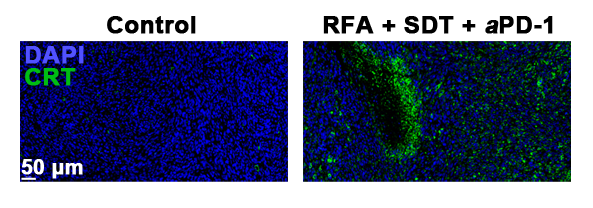

Supplement: Supplementary Figure 6 — Immunofluorescence images of CRT in primary tumors of the control group and combination treatment group (scale bar: 50 μm). [file Image_6.tif]

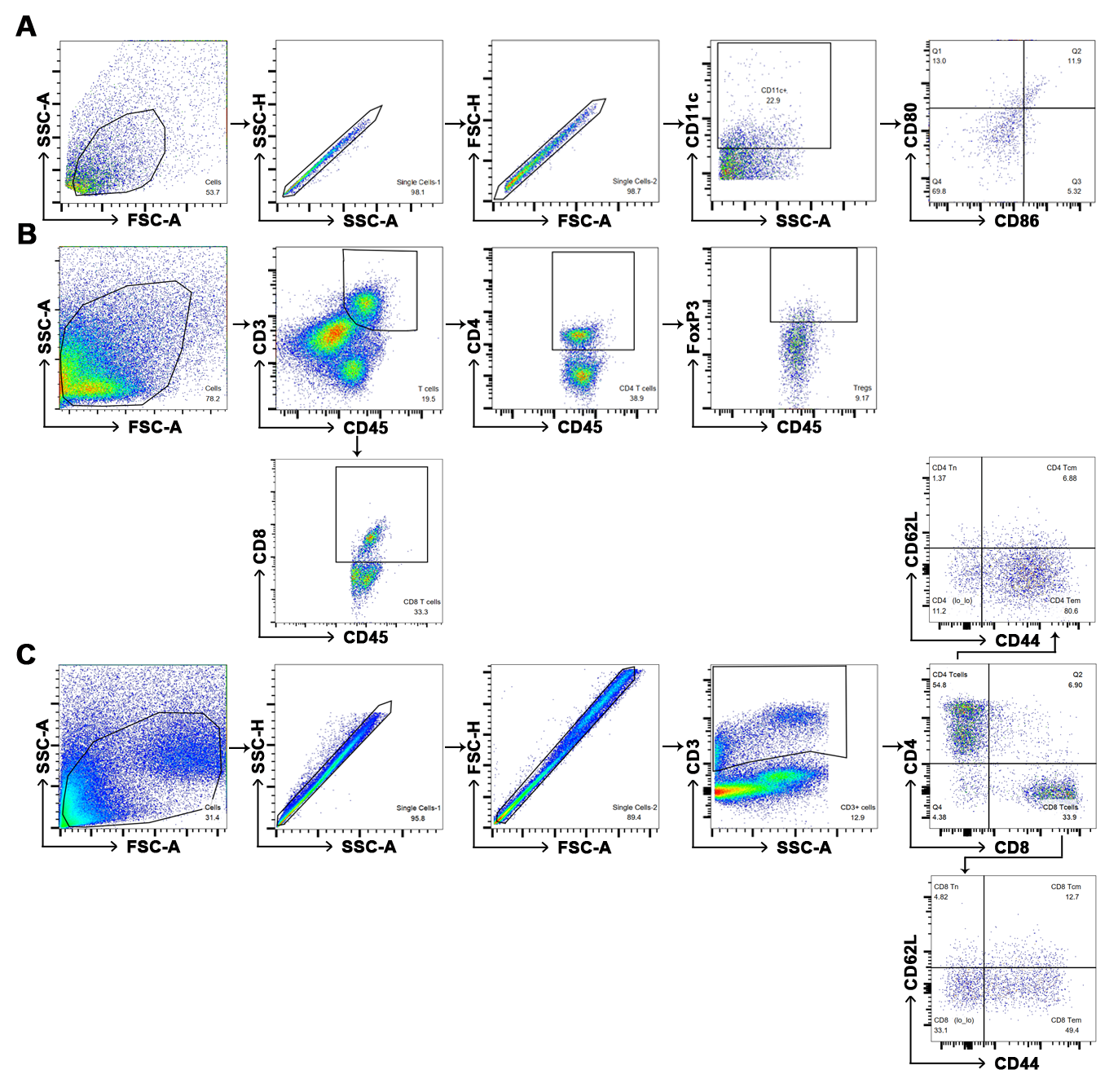

Supplement: Supplementary Figure 7 — Gating strategies for flow cytometry analyses of immune cells. (A) Gating strategy to determine the percentage of mature DCs in CD11c+ DCs, as shown in Figure 5A. (B) Gating strategy to determine the percentage of T subsets in CD45+ immune cells, as displayed in Figures 5B-G and Figures 6A-E. (C) Gating strategy to determine the percentage of Tcm in CD8+ T cells or CD4+ T cells, as shown in Figures 6F-H. [file Image_7.tif]

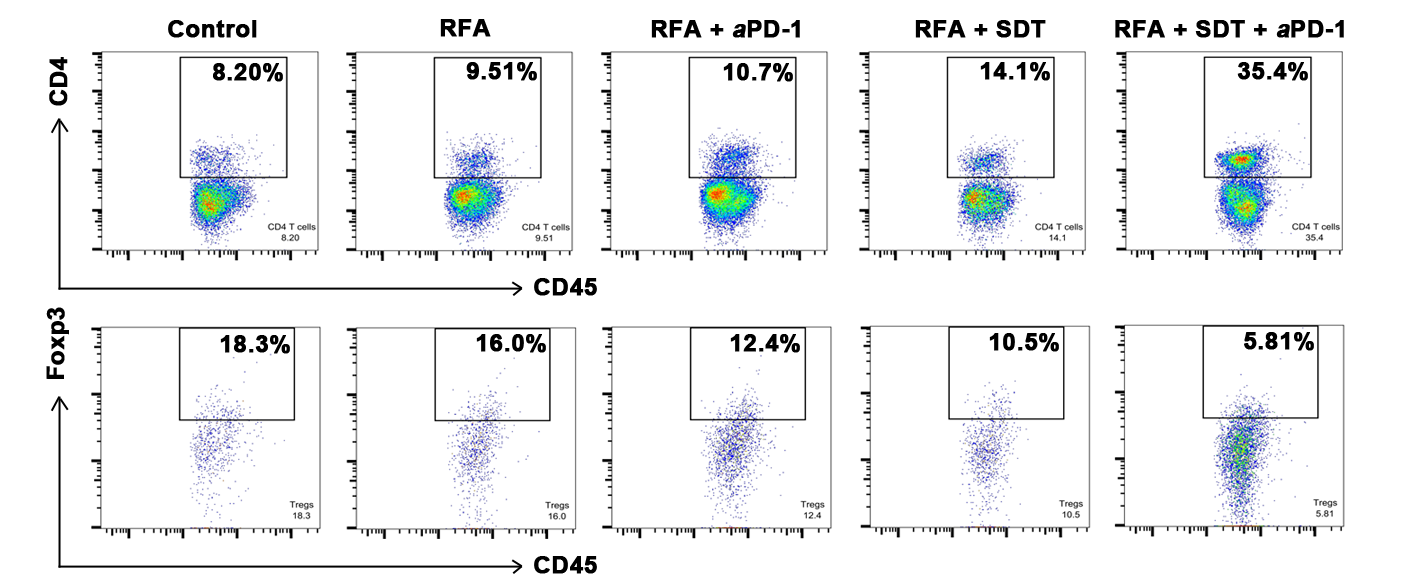

Supplement: Supplementary Figure 8 — The representative flow cytometric plots of CD4+ T cells and Tregs in distant tumors receiving different treatments. [file Image_8.tif]
